# Label–free quantitative urinary proteomics for non-invasive biomarker discovery in endometrial cancer

**DOI:** 10.3389/fmed.2026.1759839

**Published:** 2026-04-09

**Authors:** Khalid Akkour, Mohamed Rafiullah, Afshan Masood, Ibrahim O. Alanazi, Assim A. Alfadda, Salini Scaria Joy, Ali Bassi, Hani Alhalal, Eman Alshehri, Maria Arafah, Shahid Nawaz, Hicham Benabdelkamel

**Affiliations:** 1 Obstetrics and Gynecology Department, College of Medicine, King Saud University, Riyadh, Saudi Arabia; 2 Strategic Center for Diabetes Research, College of Medicine, King Saud University, Riyadh, Saudi Arabia; 3 Proteomics Resource Unit, Obesity Research Center, College of Medicine, King Saud University, Riyadh, Saudi Arabia; 4 Healthy Aging Research Institute, King Abdulaziz City for Science and Technology (KACST), Riyadh, Saudi Arabia; 5 Department of Medicine, College of Medicine, King Saud University Medical City, King Saud University, Riyadh, Saudi Arabia; 6 Obstetrics and Gynecology Department, King Saud University Medical City, King Saud University, Riyadh, Saudi Arabia; 7 Department of Pathology, College of Medicine, King Saud University, King Saud University Medical City, Riyadh, Saudi Arabia

**Keywords:** biomarker discovery, endometrial cancer, gynecologic oncology, non-invasive diagnosis, urinary proteomics

## Abstract

**Background:**

Endometrial cancer (EC) is a growing global healthcare concern. The diagnosis of EC involves invasive techniques. There is a pressing need for reliable, non-invasive screening methods utilizing biomarkers that diagnose these patients.

**Methods:**

We conducted a case–control study consists of 40 women including 20 EC (cancer group) and 20 controls (control group) recruited from King Khalid University Hospital, King Saud University. All participants include both cancer and control groups underwent histopathological examination for confirmation. Cancer group had a confirmed diagnosis of EC and control group confirmed benign tissue, verified by histopathological examination. Midstream urine samples were collected under standardized fasting conditions, and proteins extracted using methanol–chloroform precipitation. An untargeted label-free LC-MS/MS mass spectrometric approach combined with bioinformatics was used to determine changes in the proteomic profiles. Multivariate statistical analysis (PCA, OPLS-DA) using MetaboAnalyst v6.0. functional annotation and pathway enrichment were carried out using Ingenuity Pathway Analysis (IPA) and PANTHER classification to identify key biological processes and canonical pathways associated with EC.

**Results:**

The participants in this cohort were matched for age, with a mean of 59.50 ± 7.13 years in the EC group, and 54.15 ± 10.45 years, in the controls. The study found EC patients had significant differences in 193 proteins (117 upregulated and 76 downregulated) when compared to the controls. There was a clear separation seen between the EC and control groups in multivariate analyses using the PCA, PLS-DA, and OPLS-DA. Glutamate dehydrogenase 1 (GLUD1) and Iduronate 2-sulfatase, both of which were found to be downregulated, and histidine-rich glycoprotein, which was upregulated in EC patients with AUCs of 0.945, 0.965, and 0.875 in the receiver operating curve analysis. Pathway enrichment analysis and network analysis provided molecular insights into the activation of Fc gamma receptor-dependent phagocytosis, complement activation, TRIM21 signaling, and amino acid metabolism.

**Conclusion:**

The study identified three key biomarkers, GLUD1, Iduronate 2-sulfatase, and histidine-rich glycoprotein, that were significantly dysregulated in patients with EC. The findings highlight that there are systemic immune engagement and tumor-driven metabolic shifts in EC.

## 1 Introduction

Endometrial cancer (EC) is a growing global healthcare concern, with over 414,000 new cases and 90,000 deaths in 2021, making it the fourth most newly diagnosed cancer in women and sixth in cancer-related mortality (1). The burden was seen more pronounced among women aged 50 years and older. The increasing incidents of EC are linked to obesity, metabolic syndrome, and aging populations (2). In Saudi Arabia, EC is ranked fourth with 6.2% of all cancer cases diagnosed among Saudi females (3). The most common type of EC is type 1, which is a relatively low-grade malignancy. The risk of EC is increased in the continuously elevated estrogenic environment without progestins. Conditions that enhance an estrogenic climate, such as estrogen-secreting tumors, polycystic ovaries that interfere with ovulation, and menstrual cycles, are associated with a high risk of EC (4).

The diagnosis of EC includes techniques such as transvaginal ultrasound, hysteroscopy, and endometrial biopsy. Ultrasound is widely used as an initial strategy due to its easy availability; however, it is non-specific and leads to many false-positive findings that necessitate invasive follow-up procedures (5). Consequently, many women with benign conditions undergo unnecessary invasive testing, causing patient discomfort, anxiety, and procedural risks, and also a significant healthcare and financial burden. Thus, there is a pressing need for a reliable, non-invasive screening tool that can help identify patients who will most likely benefit from a confirmatory biopsy. Urinary biomarkers can offer a promising solution to overcome this challenge. A simple, urine-based test that could discriminate EC from other benign conditions will greatly improve patient care and decrease the healthcare burden.

Urine is a non-invasive, easily obtainable biofluid that reflects systemic physiological and pathological changes, including those driven by tumor biology. It is also likely to contain uterine-derived biomarkers resulting from the contamination of the voided urine. Proteomic profiling of urine has been attempted in many cancers. Urinary proteomics was used to investigate potential biomarkers in bladder cancer. As urine is directly in contact with the bladder tissue during storage, urine is most likely to contain proteins shed from the cancer tissue in the bladder. The study found proteins associated with proliferation and necrosis of cancer cells (6). Another study analyzed the pre-existing urinary proteomics data using subtractive analysis techniques to identify 44 unique proteins from the cancer patients. This study found 14 proteins that were not previously linked to cancer (7). Machine learning was used to identify biomarkers from urinary proteomics to differentiate lung cancers from normal controls and other cancer types. Researchers developed a combinatorial model using five urinary biomarkers for early detection of lung cancers (8). Three potential biomarkers were identified from urine and validated for screening breast cancer using label-free LC-MS/MS-based proteomics (9). In EC, there is emerging evidence to show that urinary proteins can be utilized to diagnose potential cases. Njoku et al. identified urinary proteins such as SERPINA1, LRG1, and CDH1 that differentiate EC from benign conditions with high diagnostic accuracy (10). In a metabolome analysis of urine and serum in EC patients, the urine panel was able to identify EC from controls and also distinguished the two types of EC (11). Despite the urgent need, not many studies were conducted to explore the urinary proteomics of EC. In the present study, we conducted high-resolution LC-MS/MS profiling of urine samples from EC patients and matched controls, aiming to identify robust biomarkers and elucidate disease-associated pathways. By integrating differential abundance analysis with pathway analysis, we sought to uncover mechanistically relevant urinary proteomic signatures in EC.

## 2 Material and Methods

### 2.1 Ethical considerations and informed consent

This study adhered to the highest ethical standards, following both the Declaration of Helsinki and the International Conference on Harmonization Good Clinical Practice guidelines. The study protocol E-193622 received full approval from the Institutional Review Board, College of Medicine, King Saud University. Importantly, written informed consent was obtained from all participants before their involvement in the study.

### 2.2 Study participants and recruitment

This study recruited a total of 40 women, aged 46 to 75, from the outpatient clinics of the Obstetrics and Gynecology-Oncology Department at King Khalid University Hospital, King Saud University. All participants provided informed consent. The women were divided into two groups EC (cancer) group (*n* = 20; age 59.50 ± 7.13years): These participants had a confirmed diagnosis of EC, verified by histopathological examination. control group (*n* = 20; age 54.15 ± 10.45 years): The control group comprised individuals undergoing routine clinical evaluations who subsequently underwent surgical intervention for non-oncological indications. This control group provided a baseline of histologically confirmed benign tissue for comparison against the oncologicalcohort. All participants include both cancer and control groups underwent histopathological examination for confirmation. To maintain a clear proteomic focus and minimize hormonal or systemic interference, women who were fertile or had a history of any other prior cancer diagnosis were excluded.

### 2.3 Data and urine collection

After an overnight fast, 5 ml of venous blood sample were collected from each subject in a plain tube. Serum was separated and stored immediately at – 20 °C for further analysis. Following a 10-h fast, participants provided 50–100 ml of first morning midstream spot urine samples in sterile containers. This standardized collection protocol was utilized to ensure the samples were concentrated and consistent, thereby reducing the impact of hydration-induced dilution and minimizing variability unrelated to disease status. To prevent contamination and proteolysis, samples were immediately transported on ice. Urine test strips (Combur10 Test, Roche, Basel, Switzerland) were used to screen for urinary protein, infection, sugar, or occult blood. Within 30 min of collection, samples underwent centrifugation at 4,000 rpm and 4 °C for 10 min to remove insoluble material and avoid artifact-related protein release. Five ml of the resulting supernatant was then carefully aliquoted and stored at−80 °C for long-term storage. Detailed characteristics of all study participants can be found in Supplementary Table S1.

For all subjects, clinical data were collected including age, systolic blood pressure (SBP), and diastolic blood pressure (DBP). Demographic data such as body mass index (BMI) were calculated as the quotient of weight (kg) divided by height squared (m^2^). The biochemical assessments including FBG, HbA1c, serum creatinine, blood urea nitrogen were analyzed using routine laboratory procedures. eGFR was estimated using CKD-EPI creatinine equation (12).

### 2.4 Urine protein extraction and processing

Protein isolation from urine samples was performed using a methanol-chloroform precipitation method as previously described (13). Briefly, 3 ml of each sample was combined with 3 ml of methanol and 0.75 ml of chloroform. The mixture was then centrifuged at 5,000 x g for 5 min, and the upper phase was carefully discarded. Three volumes of methanol (relative to the initial sample volume) were added, and the sample was mixed and subjected to a second centrifugation at 16,000 x g for 10 min to pellet the protein. The isolated protein pellet was subsequently dried at room temperature using a vacuum centrifuge (Eppendorf Concentrator plus TM, Hamburg, Germany). The dried protein was reconstituted overnight at 4 °C in 2-fold lysis buffer pH 8.8, containing 30 mM Tris-HCl, 7 M urea, 2 M thiourea, 4% CHAPS, and 1x protease inhibitor mix). Complete protein dissolution was ensured the following day by sonication and vortexing. Finally, the protein concentration for each sample was determined in triplicate using the 2D-Quant Kit (GE Healthcare, Piscataway, NJ, USA) (14).

Fifty micrograms of urine proteins were denatured in 10 μl of 6M urea buffer. Protein disulfide bonds were reduced by adding 1 μl of 200 mM dithiothreitol (DTT) and incubating at 60 °C for 30 min. Alkylation was performed by adding 1 μl of 400 mM iodoacetamide (IAA) and incubating for an additional 30 min at room temperature in the dark. The samples were subsequently diluted with 65μl of 50 mM ammonium bicarbonate buffer and subjected to overnight digestion at 37 °C with 2.5 μl of sequencing grade modified Trypsin (Promega Corporation, Wisconsin, USA). Digestion was quenched by acidification with 7 μL of 10% formic acid (FA). The resulting peptides were desalted using Pierce C18 spin columns (Thermo Scientific, Rockford, IL, USA) and dried via vacuum centrifugation (Eppendorf Concentrator plus TM, Hamburg, Germany). Finally, the peptide concentration was determined using a Pierce Quantitative Colorimetric Peptide Assay (Thermo Scientific, IL, USA) (15–17).

### 2.5 Liquid chromatography coupled to tandem mass spectrometry (LC-MS/MS)

Peptides were reconstituted in a solution of 0.1% (v/v) FA. A 1 μl aliquot of each sample was injected onto a Dionex UltiMate 3,000 nano-LC system equipped with a WPS-3,000 autosampler (DIOnex Softron GmbH, Germering, Germany). The peptides were initially trapped and concentrated on a PepMap100 C18 trap column [3 μm, 100 Å, 75 μm 285 inner diameter (i.d.) × 20mm, nanoViper; Thermo Scientific Rockford, IL, USA] pre-equilibrated with 0.05% TFA in water, utilizing a selective trapping nano-LC setting for high-capacity sample loading. Subsequent separation was achieved using an analytical column PepMap™ C18, 50 cm × 75 μm at a flow rate of 300 nL/min. Mobile phase A consisted of 0.1%(v/v) FA in water, and mobile phase B contained 0.1% (v/v) FA and 80% (v/v) acetonitrile in water. The separation gradient began with 5% mobile phase B, increasing to 22.5% B over 139 min, and then to 45% B over 184 min. Eluting peptides were introduced into a Q Exactive Plus Hybrid Quadrupole-Orbitrap mass spectrometer (Thermo Fisher Scientific, Waltham, MA, USA) via a nanospray ion source operating in positive ion mode with a nano electrospray (nESI) potential of 2,000 V and a maximal duty cycle of 3s. Mass spectra were acquired in a data-dependent acquisition (DDA) mode. The primary mass spectrometry (MS) scan range was set to 375–1,650 m/z with a resolution of 70,000, and the secondary mass spectrometry (MS/MS) scan resolution was set to 17,500 with a fixed starting point of 80 m/z. The dynamic exclusion time was set to 20 s, and the automatic gain control (AGC) targets were set to 3 × 10^6^ and 1 × 10^5^ for MS and MS/MS scans, respectively (15–17).

### 2.6 Data processing

Raw MS and MS/MS data were processed using Proteome Discoverer v3.0 (Thermo Fisher Scientific), employing Sequest as the search engine against the HUMAN-refprot-isoforms.fasta sequence database. The search parameters included: (1) a precursor/fragment mass tolerance of 15 ppm/0.02 Da, respectively; (2) a maximum of two missed cleavages; (3) trypsin (Full) as the enzyme; (4) dynamic modifications of peptide N-terminal acetylation and methionine oxidation; and (5) the static modification of cysteine carbamidomethylation. Peptide-spectrum matches (PSMs) were rigorously filtered, requiring protein and peptide FDRs of 1% and a minimum of two unique peptides per protein. Only proteins identified and quantified via Label-free quantification (LFQ) intensity were used for subsequent analyses. The resulting list of quantified proteins, exported from Proteome Discoverer and manually formatted, underwent further non-redundant filtration (FC ≥1.5 for upregulation and ≤ 0.67 for downregulation; *P* ≤ 0.05), peak picking, and missing value removal. The final protein set was then subjected to multivariate statistical analysis using MetaboAnalyst v. 6.0 (http://www.metaboanalyst.ca), (McGill University, Montreal, QC, Canada) (http://www.metaboanalyst.ca, accessed on 15 June 2025) following median normalization, Pareto scaling, and log transformation (18). Principal Component Analysis (PCA) was performed for visualization of study groups and outlier detection, and an Orthogonal Partial Least Squares Discriminant Analysis (OPLS-DA) model was also generated for supervised and unsupervised models. To ensure the statistical validity and predictive reliability of the supervised multivariate analysis, the OPLS-DA model was further evaluate using a 5-fold internal cross-validation to determine goodness of fit (R2Y) and predictive ability (Q2), and a 100-cycle permutation test was conducted to confirm that the observed separation was not due to random chance (15–17).

### 2.7 Statistical and bioinformatics analysis

Demographic and clinical parameters are presented as a mean ± standard deviation. Comparisons of two groups were performed using the Student's *t* test. A P < 0.05 was considered statistically significant. All statistical analyses were performed using SPSS (v.26.0; IBM Corp., Armonk, NY, USA). Statistical analysis was conducted using a Student's *t*-test implemented in Proteome Discoverer v3.0. Adjusted *P*-values (*q*-values) determined by the False Discovery Rate (FDR) method were considered significant for values below 0.01. Proteins demonstrating differential expression were selected based on the criteria of *P*-value ≤ 0.05 and FC ≥1.5 for up regulation and FC ≤ 0.67 for down regulation, and the resulting data were exported from Proteome Discoverer. For functional and pathway analysis, the quantitative data were imported into the Ingenuity Pathway Analysis (IPA) software (Ingenuity Systems, http://www.ingenuity.com, accessed on 09 July 2025; QIAGEN Inc., Hilden, Germany), which determined associated functions and pathways by integrating experimental expression data with known molecular interaction networks. Furthermore, the PANTHER (Protein Analysis Through Evolutionary Relationships) classification system (http://www.pantherdb.org, accessed on 04 September 2025) was used to categorize the identified proteins according to their molecular function and biological process. The complete urinary proteomics pipeline for EC biomarker identification is outlined in Supplementary Figure S1.

## 3 Results

### 3.1 Characteristics of study participants

Supplementary Table S1 outlines the characteristics of study subjects categorized by EC stage and grade. The demographic, clinical, and biochemical characteristics of study participants are summarized in Supplementary Table S2. There were no statistically significant differences between the control and EC groups regarding age and blood pressure. Despite both cohorts falling within the obese category, the groups were well-matched, with no statistically significant difference in BMI observed between them (*P* = 0.259). Renal function markers, including serum creatinine, BUN and eGFR, were comparable across both cohorts. Both groups consisted of individuals in the early stages of diabetes who were not currently taking any anti-diabetic medications, providing a clear baseline for observation. The fasting blood glucose (FBG) levels were slightly higher in the EC group compared to the control group, though this difference was not statistically significant (*P* = 0.535). Similarly, the HbA1c levels for the EC group were lower than the Control group, but this also failed to reach statistical significance (*P* = 0.093). These results indicate that despite individual variations typical of early-stage diabetes, both groups were well-matched regarding their glycemic profile prior to intervention.

### 3.2 Label-free quantitative proteomics analysis

The label-free quantitative proteomics to compare protein expression between cancer and control groups, successfully quantifying 2,662 non-redundant proteins after necessary processing steps (unique peptide identification, filtration, peak picking, and missing value removal). Based on the defined threshold for differential expression (FC ≥1.5 for up regulation and FC ≤ 0.67 for down regulation and *P*-value ≤ 0.05), we found 193 proteins to be significantly regulated in the cancer (Supplementary Table S3).

### 3.3 . Differentially expressed protein identification

**Figure 1:**
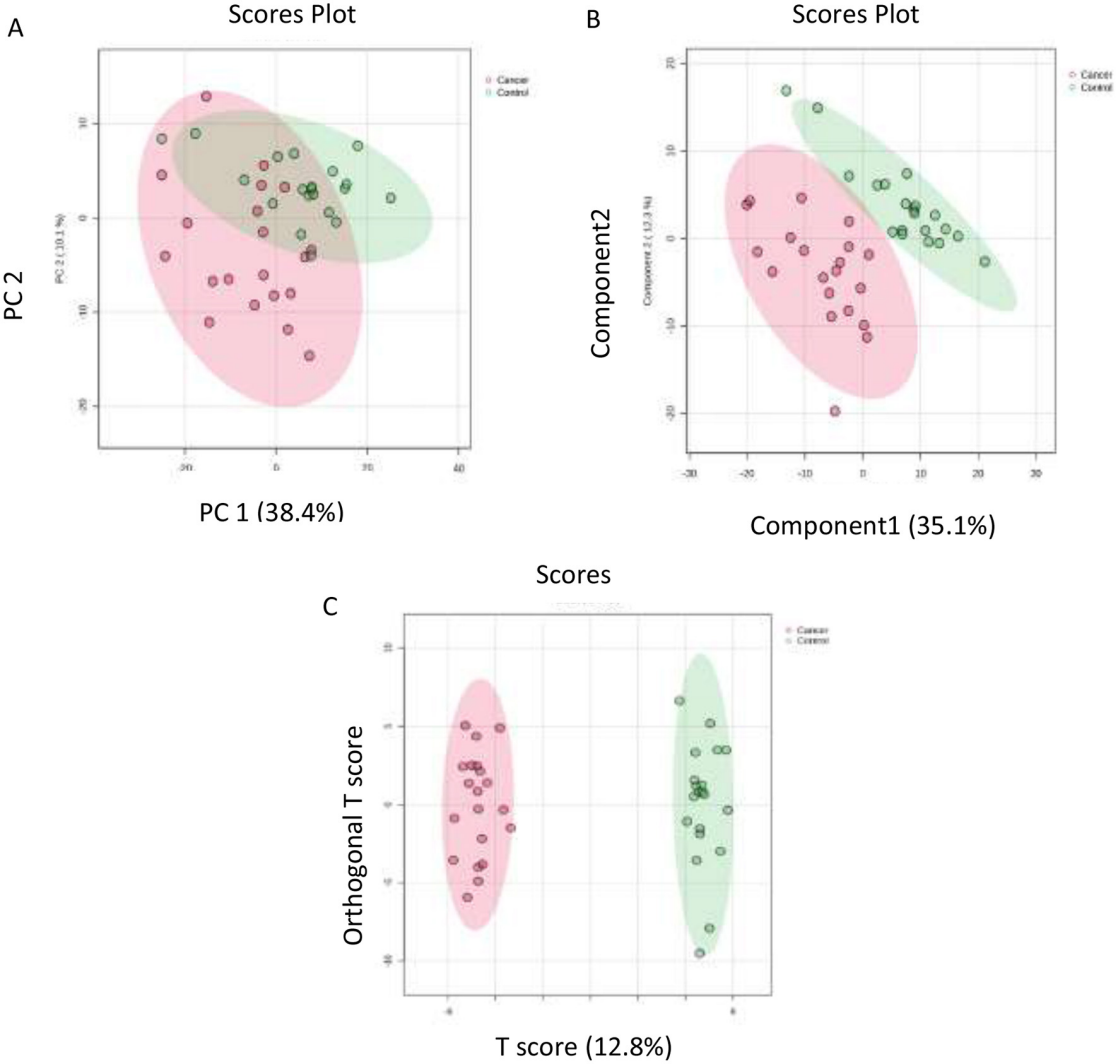
Multivariate statistical analysis of proteomic data **(A)** Principal Component Analysis (PCA) plot illustrates the distribution of samples based on the two principal components. **(B)** Partial Least Squares Discriminant Analysis (PLS-DA) demonstrates a clear separation between the cancer and control groups. **(C)** Orthogonal Partial Least Squares Discriminant Analysis (OPLS-DA) shows a distinct separation between the two groups, confirming a significant proteomic difference between the cancer and control conditions. The robustness of the created models was evaluated by the fitness of the model (R2Y = 0.941) and predictive ability (Q2 = 0.802) values in a larger dataset (*n* = 100).

To visualize and classify the distinct proteomic profiles between the control and cancer groups, we employed a combination of unsupervised and supervised multivariate analyses. A natural clustering through unsupervised principal component analysis (PCA) followed by partial least squares discriminant analysis (PLS-DA) and Orthogonal PLS-DA (OPLS-DA) and confirming model stability through rigorous cross-validation and permutation testing, provide a high degree of confidence in the statistical validity and reliability of the identified urinary proteomic signatures (Supplementary Figure S2). The unsupervised model demonstrated a clear, evident, and significant separation between the two groups (R2Y = 0.992 and Q2 = 0.853), indicating variations in proteomic expression between EC and control Supplementary Figure S2. In supervised multivariate analyses, PCA to observe natural groupings, followed by partial least squares discriminant analysis (PLS-DA) and Orthogonal PLS-DA (OPLS-DA) to maximize group separation based on proteins meeting our selection criteria (*P*-value ≤ 0.05 and FC ≥1.5 for upregulation and ≤ 0.67 for downregulation) Figure 1. The model demonstrated a clear, evident, and significant separation between the two groups (R2 = 0.941 and Q2 = 0.802), indicating variations in proteomic expression between EC and control Supplementary Figure S3.

**Figure 2:**
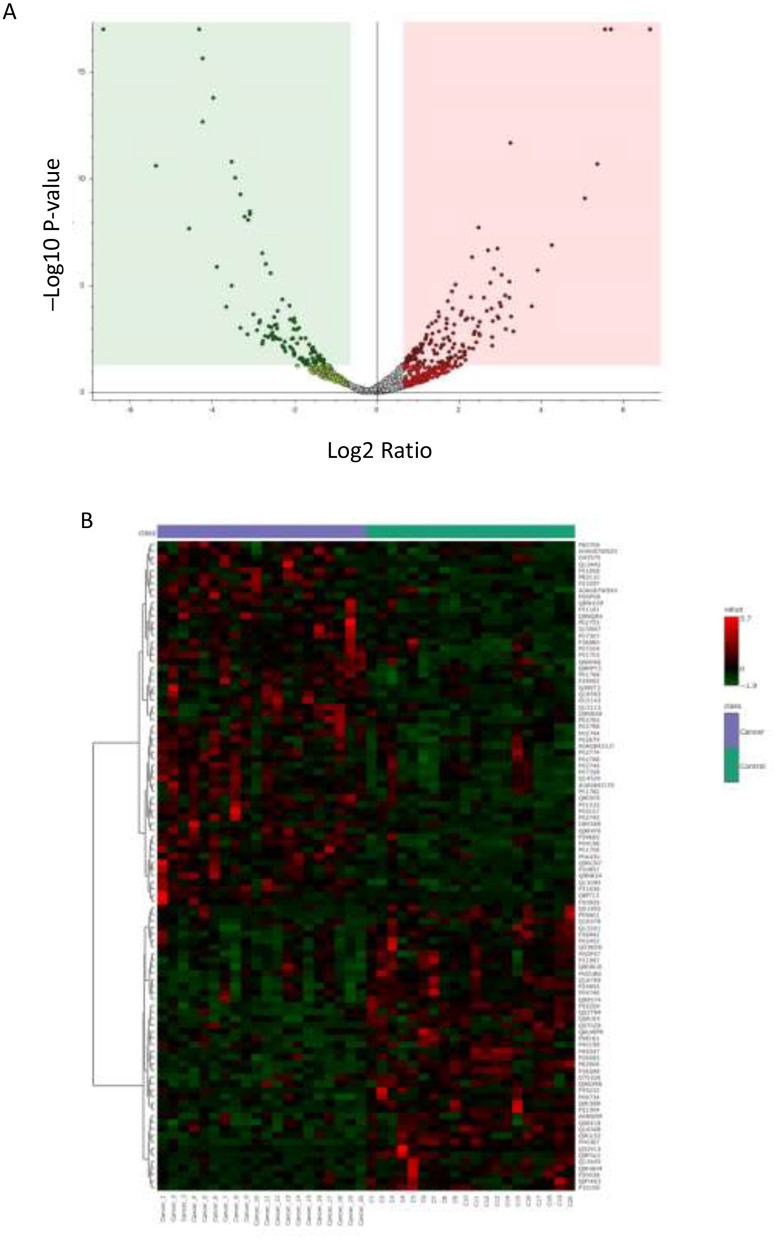
Identification of differentially expressed proteins **(A)** Volcano Plot displays the differential expression of urine proteins between the endometrial cancer (cancer) and control groups. Proteins with a statistically significant change (unpaired *t*-test, *P*-value ≤ 0.05) and a substantial difference in abundance fold change FC ≥1.5 for upregulation and ≤ 0.67 for downregulation) are highlighted. Red dots represent upregulated proteins, green dots represent downregulated proteins, and gray dots indicate proteins that are statistically non-significant. **(B)** Hierarchical Clustering (HAC) and Heat Map Analysis shows clear separation and clustering of samples, with the color range bar indicating downregulated proteins as green and upregulated proteins as red.

Analysis using a volcano plot (*P*-value ≤ 0.05, FC ≥1.5 for upregulation and ≤ 0.67 for downregulation) revealed 193 significantly dysregulated proteins in cancer, comprising 117 upregulated and 76 downregulated proteins (Figure 2A). This differential expression is clearly represented in the heat map (Figure 2B), indicating that these proteins are strong candidates for use as biomarkers to track the molecular and cellular changes associated with cancer (Supplementary Table S3).

### 3.4 . Evaluation of potential protein biomarkers

**Figure 3:**
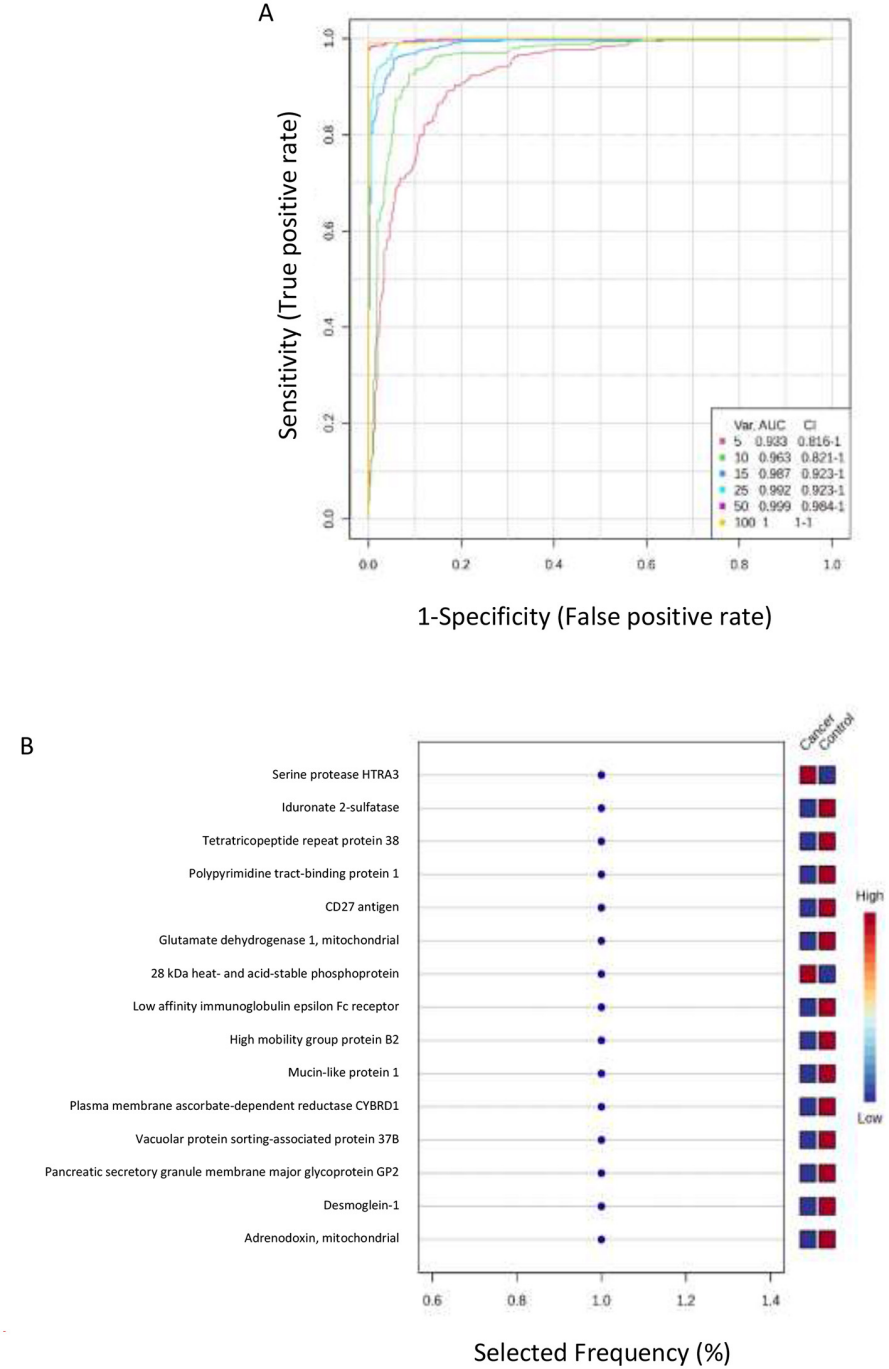
Biomarker evaluation and selection. **(A)** Receiver Operating Characteristic (ROC) curves derived from the PLS-DA model. The plot illustrates the diagnostic performance in distinguishing endometrial cancer from the control group using panels of increasing size. The Area Under the Curve (AUC) and 95% Confidence Intervals (CI) are provided for models incorporating the top 5, 10, 15, 25, 50, and 100 protein features, demonstrating the relationship between feature number and predictive accuracy. **(B)** Frequency plot shows the top 15 significantly dysregulated identified protein biomarkers when comparing the endometrial cancer (cancer) and control groups, highlighting the most consistently altered proteins.

The potential of the identified proteins to serve as biomarkers was assessed using the ROC curve analysis. We employed partial least squares discriminant analysis (PLS-DA) as a method for both classification and feature ranking to conduct a multivariate exploratory ROC analysis. Six features in the ROC curve obtained from PLS-DA and cross-validation (CV) had an area under the curve (AUC). The ROC curve showed a set of variant proteins (5, 10, 15, 25, 50, and 100), with different AUCs and confidence intervals (CIs) (Figure 3A).

Further analysis (Figure 3B) revealed the top 15 significantly dysregulated proteins when comparing control and cancer groups. Proteins upregulated in cancer include Serine protease HTRA3 and 28 kDa heat- and acid-stable phosphoprotein. Conversely, the following were downregulated: Iduronate 2-sulfatase, Tetratricopeptide repeat protein 38, Polypyrimidine tract-binding protein 1, CD27 antigen, Glutamate dehydrogenase 1 (mitochondrial), Low affinity immunoglobulin epsilon Fc receptor, High mobility group protein B2, Mucin-like protein 1, Plasma membrane ascorbate-dependent reductase CYBRD1, Vacuolar protein sorting-associated protein 37B, Pancreatic secretory granule membrane major glycoprotein GP2, Desmoglein-1 and Adrenodoxin (mitochondrial).

**Figure 4:**
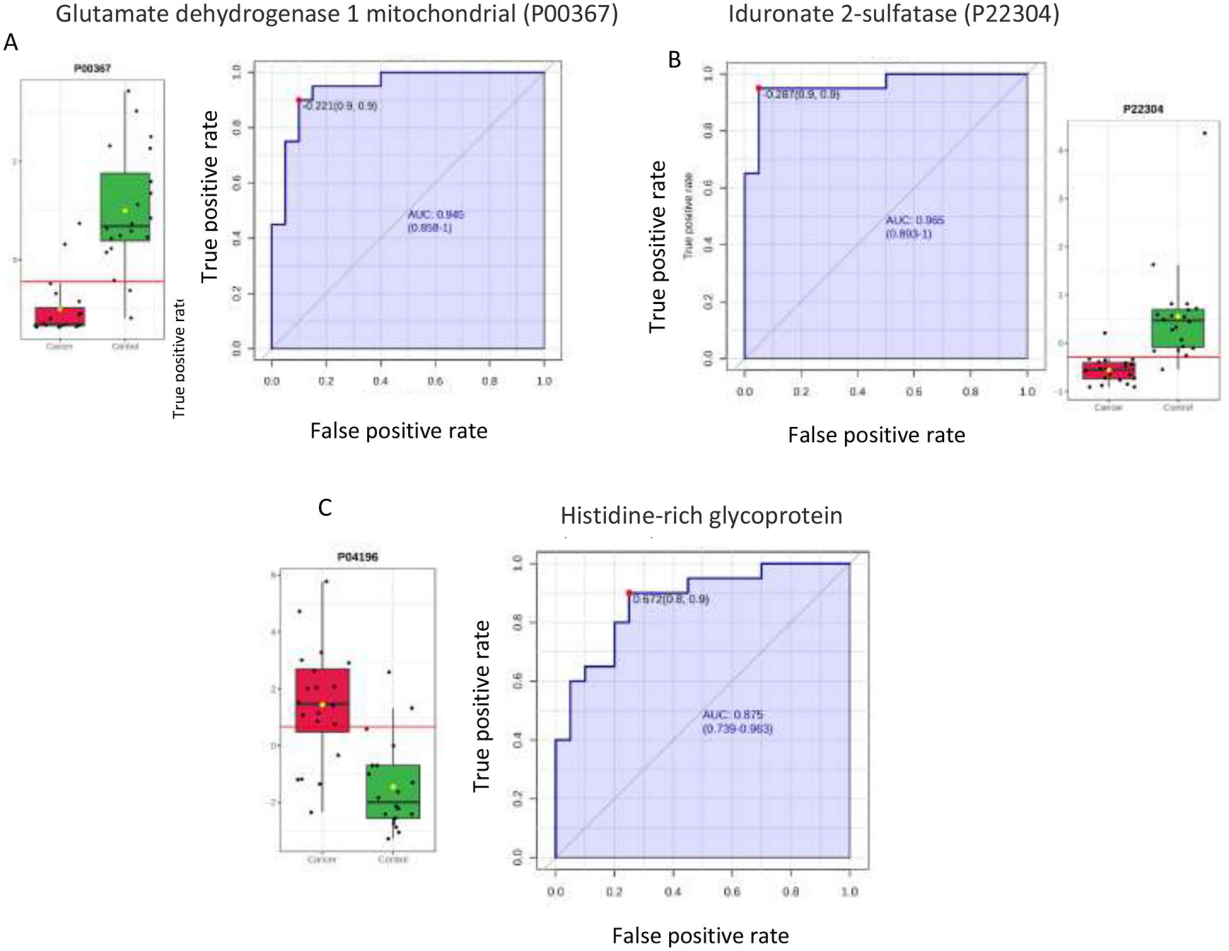
Box and whisker plots illustrating the most significantly dysregulated proteins capable of distinguishing between endometrial cancer (cancer) and Control group, based on their high Area Under the Curve (AUC) values. Cancer patients are represented by red, while control are represented by green. A red line within each box plot indicates the optimal cutoff point for the biomarker, designed to maximize both sensitivity and specificity. **(A)** Glutamate dehydrogenase 1, mitochondrial (P00367), as the downregulated protein in the cancer group (AUC = 0.945), showing a significant difference (*P* ≤ 0.05) and a fold change ≤ 0.67. **(B)** Iduronate 2-sulfatase (P22304), the top downregulated protein in the cancer group (AUC = 0.965), significant difference (*P* ≤ 0.05) and a fold change ≤ 0.67. **(C)** Histidine-rich glycoprotein (P04196), the upregulated protein in the cancer group (AUC = 0.875), demonstrating a significant difference (*P* ≤ 0.05 and a fold change ≥ 1.5).

The diagnostic potential of four key proteins distinguishing the cancer and control groups was individually confirmed using ROC curve analysis and detailed expression plots. Two proteins were found to be significantly downregulated in the cancer group: glutamate dehydrogenase 1, mitochondrial (P00367) and iduronate 2-sulfatase (P22304), which demonstrated high predictive power with AUC values 0.945 and 0.965, respectively (Figures 4A, B). Conversely, protein was significantly upregulated in cancer, histidine-rich glycoprotein (P04196), achieving strong diagnostic separation with AUC values of 0.875 (Figure 4C). Box-and-whisker plots confirmed the decreased expression of glutamate dehydrogenase 1(mitochondrial) and iduronate 2-sulfatase and the increased expression of Histidine-rich glycoprotein solidifying their roles as reliable biomarkers for cancer.

### 3.5 Interaction network analysis of differentially expressed proteins

**Figure 5:**
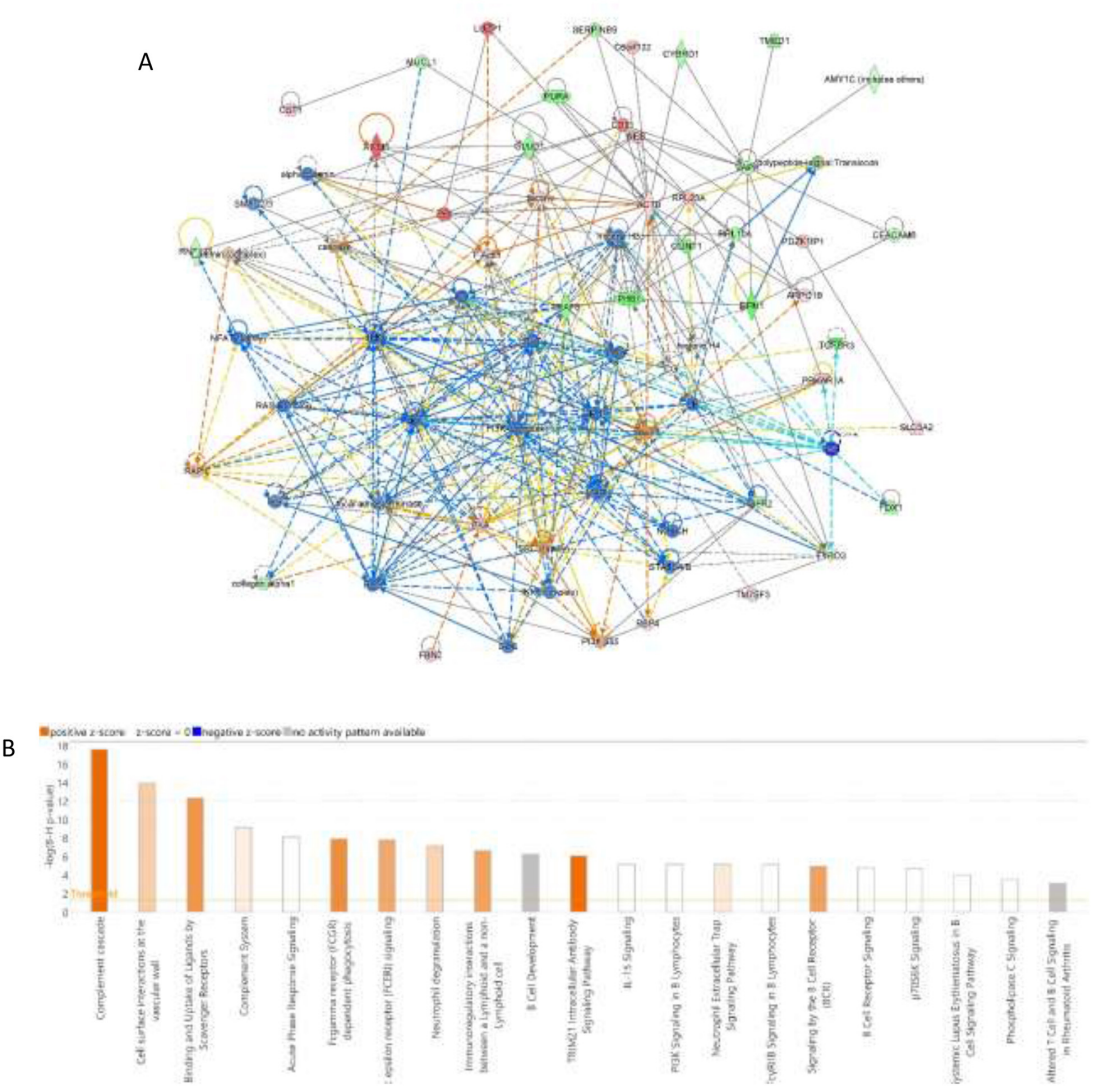
Network and pathway analysis of dysregulated protein **(A)** Schematic of Top Network Pathways presents a visual representation of the most relevant network pathways derived from the differentially regulated urine proteins in the endometrial cancer and control groups. Nodes colored blue indicate proteins that are downregulated, while orange nodes represent upregulated proteins. **(B)** Canonical Pathway Analysis (IPA) shows the results from Ingenuity Pathway Analysis (IPA) (QIAGEN Inc., Hilden, Germany), which identified and ranked the most significant canonical pathways based on their *P*-value.

The biological impact resulting from the changes in protein abundances within the dataset was investigated using IPA software. This software generates a protein-protein interaction network by calculating a score based on the optimal fit between the input dataset of differentially regulated proteins and the biological functions database. The resulting network, which visually maps these protein interactions, is presented in Figure 5. The IPA identified cancer, cellular development, cellular growth and proliferation as the network pathways affected with the highest score between EC and control groups (score of 66) (Figure 5A). The top canonical pathways included complement cascade, binding and uptake of ligands by scavenger receptors, TRIM21 intracellular antibody signaling pathway, Fcgamma receptor (FCGR) dependent phagocytosis with positive z-score (Figure 5B).

### 3.6 Protein analysis through evolutionary relationships (PANTHER) classification

**Figure 6:**
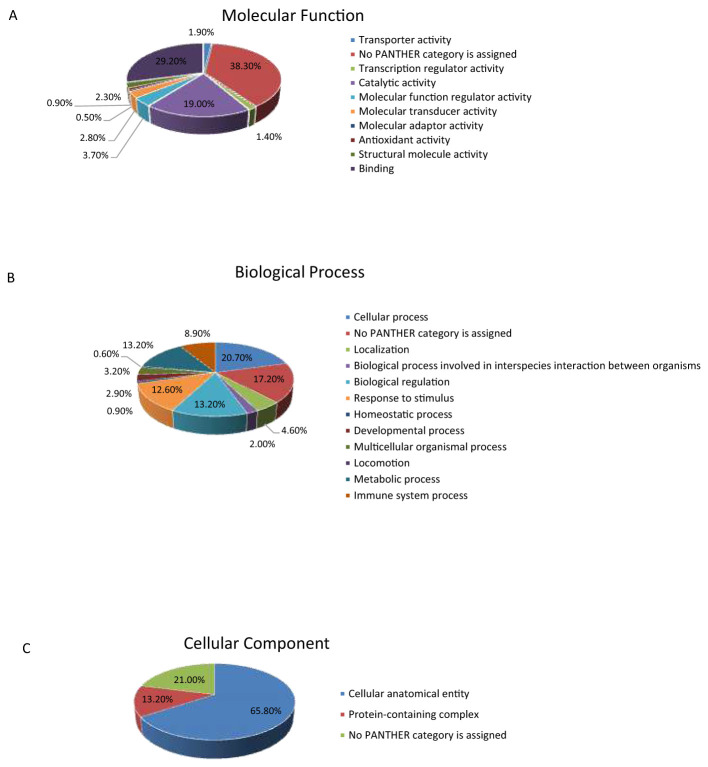
Comparative visualization of the identified proteins categorized by the PANTHER classification system into their respective **(A)** molecular functions, **(B)** biological processes, and **(C)** cellular components.

The PANTHER classification system was utilized to categorize the differentially expressed proteins based on their molecular function, biological processes, and cellular components. Analysis of molecular functions (Figure 6A) revealed that the majority of the proteins identified were enzymes primarily involved in binding (29.2%) and catalytic activity (19.0 %). Regarding biological processes (Figure 6B), the dominant categories were cellular processes (20.70%) and metabolic processes and biological regulation (13.2%). Finally, classification by cellular components (Figure 6C) showed that most proteins were localized to the cellular anatomical entity (65.8%), with the protein containing complex (13.2%) as the next most prominent location.

## 4 Discussion

The findings suggest that urinary proteomics hold a potential as a non-invasive technique for detecting EC. Among the 193 dysregulated proteins in patients with EC, 117 were upregulated and 76 were downregulated when compared to the controls. The clear separation seen between the two groups in multivariate analyses using the PCA, PLS-DA, and OPLS-DA confirms that the EC patients have a distinct urinary proteomic profile. The PLS-DA model showed strong separation and identified candidate biomarkers. The OPLS-DA model further reinforced the confidence by showing a total group separation, as it reduced the noise and enhanced focus on the proteins. It also supported the selection of the robust candidate biomarkers. It is further evident from the very high AUC values (>0.933) from the ROC curves generated using the OPLS-DA model by combining the proteins.

Glutamate dehydrogenase 1 (GLUD1) and Iduronate 2-sulfatase, both of which were found to be in lower abundance, and histidine-rich glycoprotein in higher abundance in EC patients. They exhibited an AUC ≥ 0.80, which is considered a strong threshold for clinical usefulness, reflecting high sensitivity and specificity in distinguishing disease from control states (19). This finding suggests that these proteins are the potential candidates for evaluation as urinary biomarkers for ECs in larger cohorts. These candidate biomarkers also have biological relevance in the ECs. GLUD1 is a mitochondrial enzyme involved in the anaplerosis for the TCA cycle. It catalyzes the oxidative deamination of glutamate to α-ketoglutarate, an important substrate in the TCA cycle. It is usually upregulated in glutamate-dependent cancers (20). The possible downregulation of GLUD1 in EC cells may indicate metabolic reprogramming in shifting toward glycolytic glucose-driven metabolism, a likely Warburg effect (21). The Human Protein Atlas showed a lower GLUD1 expression in the EC tissues (22).

Iduronate 2-sulfatase is a lysosomal enzyme that catalyzes the hydrolysis of sulfate groups from glycosaminoglycans such as heparan sulfate and dermatan sulfate. In our study, it was found to be significantly lower in the urine in the patients with EC in comparison to the controls. Its lower expression systemically could lead to the accumulation of dermatan sulfate proteoglycans. In breast cancer cells, iduronate 2-sulfatase was found to be depleted in the tumor epithelia. The decreased expression of Iduronate 2-sulfatase in breast cancer cells resulted in increased dermatan sulfate levels, causing collagen remodeling around cancer cells to increase their invasiveness (23). Dermatan sulfate is also reported to have tumorigenic properties. In esophageal squamous cell carcinoma, it was found to be fivefold higher than in normal tissue (24). While there are no studies in EC about the role of iduronate 2-sulfatase, the findings from the breast cancer cells study indicate that the downregulation of iduronate 2-sulfatase is likely to potentiate the invasiveness of cancer cells. The lower urinary abundance of iduronate 2-sulfatase might have been an outcome of systemic downregulation and, therefore, a likely indication of the invasiveness of the EC. The urinary Iduronate 2-sulfatase levels demonstrated excellent discriminative performance in our cohort, reinforcing its potential to be explored as a non-invasive biomarker. Given the significant dysregulation of this protein, further investigation into its role in EC pathophysiology is warranted.

Histidine-rich glycoprotein, a multifunctional protein that regulates coagulation, cell adhesion, fibrinolysis, modulates immune responses, and controls angiogenesis (25). It is primarily a blood protein produced in the liver. Histidine-rich glycoprotein exhibits a dual role in cancers. While it can suppress tumor growth under certain conditions, it may also facilitate immune evasion or vascular remodeling. A proteomic study of serum from patients with endometrial hyperplasia and carcinoma showed downregulation of histidine-rich glycoprotein (26). This study, however, focused on early-stage EC and hyperplasia, whereas our study recruited patients of EC only. It could be a reflection of tumor-induced dysregulation of the host defense proteins. The observed increase in urinary levels may indicate enhanced renal excretion, potentially accounting for the reduced serum levels documented in earlier studies. It can also be an outcome of increased systemic turnover, possibly due to the heightened inflammatory signaling, or vascular leakage. The exact role of histidine-rich glycoprotein depends on factors such as tumor type and tumor microenvironment (27). Further investigation into its role in EC and its correlation with tumor burden or stage will be required. Our results identified potential biomarker candidates. However, confirmation in a large prospective cohort and external validation using orthogonal methods like targeted proteomics and immunoassays is necessary to establish these proteins as biomarkers.

### 4.1 Proteins found in higher abundance in EC

In addition to these biomarker candidates, our study also found 193 proteins that were significantly dysregulated in EC patients. Mannosyl-oligosaccharide 1, 2-alpha-mannosidase (MAN1C1) is found in higher abundance in the urine of EC patients. Its upregulation in Glioblastoma multiforme patients was associated with poor prognosis. MAN1C1 expression was linked to disruption of the immune response in the glioblastoma tumor microenvironment (28). On the other hand, MAN1C1 was downregulated in renal cell carcinoma, and its overexpression inhibited the human clear cell renal cell carcinoma cell lines, demonstrating a potential tumor suppression role (29). In our study, the renal excretion of MAN1C1 was higher in EC patients than in controls, indicating a possible systemic overexpression and association with the poor prognosis of EC. Apoptosis-inducing factor 1 (AIF1), a mitochondrial flavin protein, induces apoptosis in response to pro-apoptotic factors. It has been implicated in several cancers. Elevated mRNA levels and protein expression have been found in many cancers, including lung cancer, pancreatic cancer, prostate cancer, colorectal carcinomas, gastric carcinomas, esophageal squamous cell carcinoma, and skin squamous cell carcinoma (30). AIF1 knockout mouse models exhibited prolongation of overall survival in lung carcinomas (31). Elevated AIF1 levels have not been reported in EC. Its increased urinary abundance in EC patients in our study is consistent with the literature on other forms of cancer.

Zyxin is another protein that was found to be highly abundant in the urine of the EC patients in our study. It is a cytoskeletal protein that regulates cell adhesion and migration. It also plays a role in cell differentiation, proliferation, and apoptosis. The Cancer Genome Atlas data show that zyxin is downregulated in EC (22). In a gene expression profiling study, the zyxin gene was found to be downregulated in EC (32). However, our results show that the urinary excretion of zyxin in EC patients was significantly higher than that of the control group. As tumor cells lose adhesion and structural integrity, zyxin and other proteins could be released into the extracellular environment, leading to an increased urinary excretion. Leucine Zipper Protein 1 is a leucine zipper motif-containing protein and functions as a dimerization domain. It is reported to be upregulated in many tumors, including breast and cervical cancers (33, 34). Our study reports the higher urinary abundance of Leucine Zipper Protein 1 in EC, reflecting a likely systemic upregulation of this protein. The results showed a higher abundance of CD81 antigen, a marker of exosomes. EC cells are known to release exosomes. Urine-derived exosomes represent a valuable source of biomarkers and have been investigated in EC (35). However, in EC, urinary exosomes may originate from either the systemic circulation or direct shedding by the uterus. Another protein, Proteasome subunit beta type-10, an immunoproteosome involved in the clearing of damaged intracellular proteins, is reported to be increased in most cancers (36). Cancer cells undergo damage to their DNA and proteins under oxidative stress. Therefore, most cancer cells show increased expression of proteosomes. Our study also found it in higher abundance of proteasome subunit beta type-10 in EC patients. Angiopoietin-related protein 3 (ANGPTL3) is found to be higher in the urine of EC patients. This protein is involved in angiogenesis and cell proliferation, two important processes for cancer growth and metastasis (37). ANGPTL7 is reported to be expressed highly in EC cells. ANGPTL3 has not been reported in EC so far. It is also linked to lipid metabolism and expressed in the liver and kidneys. Further studies are needed to clarify its role in the EC.

### 4.2 Proteins found in lower abundance in EC

Transforming growth factor β (TGFβ) is a tumor suppressor, and cancer cells evade its suppressive effect by inactivating the pathway through the mutation of its receptors, such as the transforming growth factor β receptor type 3 (TGFBRIII) (38). In early stages of tumorigenesis, TGFβ acts as a tumor suppressor by inducing cell cycle arrest and apoptosis, leading to inhibition of uncontrolled proliferation. However, in advanced stages of cancer progression, tumor cells escape from the anti-proliferative effects of TGFβ, and the high levels of TGFβ produced by the tumor cells switch to promoting progression and metastasis by enhancing cell invasion, and angiogenesis (39). There is no high TGFβ in the current study in EC patients, indicating that the cancer has not reached the level of switching the TGFβ to promote tumor progression. However, this must be interpreted with caution as we are investigating the urinary protein abundance in this study, and it may not accurately reflect the proteomic changes in the tumor microenvironment. Prohibitin 1 is an antiproliferative protein that is found to be downregulated in many cancers. However, in EC, it acts as a pro-proliferative factor. In EC tissues, prohibitin 1 is overexpressed, and it is associated with poor prognosis. Estrogen enhances EC proliferation by inducing the overexpression of prohibitin 1 (40). The lower urinary abundance of prohibitin 1 in EC patients in the current study warrants further investigation. The overexpression of prohibitin 1 is within the EC tissues, and the reduced level in urine might reflect altered protein shedding from the tissues, rather than the lack of overexpression. Dolichyl-diphosphooligosaccharide–protein glycosyltransferase subunit 1 (RPN1) is a core component of the oligosaccharyl transferase complex in the endoplasmic reticulum. It is an oncogenic driver implicated in tumor progression across many cancers, primarily through its protein modification role (41). The lower urinary abundance may be a secondary effect of altered cellular processing, despite the protein being likely to have been overexpressed in the tissue itself. Elafin, a serine protease inhibitor, protects the epithelium against pathogens. Its expression in cervical cancer was gradually lost with cancer progression (42). Therefore, the lower abundance in our study could be an outcome of EC progression. Cytosolic galectin-9 predicted a better overall prognosis in EC patients. It regulates cell adhesion and is associated with lower tumor progression and progression-free survival (43). The lower abundance of galectin-9 found in our study is a likely indicator of tumor progression in EC patients. Further investigations will be needed to utilize this protein as a biomarker for tumor progression.

### 4.3 Network analysis of biological pathways related to EC and biomarker analysis

The study found dysregulated proteins that are previously known to be associated with EC. EC cells are reported to express casein kinase II subunit alpha (CK2β). It mediates the Tumor necrosis factor (TNF)-related apoptosis-inducing ligand (TRAIL)-induced apoptosis in EC cells. Inhibition or silencing of CK2β sensitized the EC cells to TRAIL-induced apoptosis (44). Our results show that EC patients had higher urinary abundance of CK2β, indicating the presence of apoptosis-resistant EC. Mutations in fibroblast growth factor receptor 2 (FGF2) are found in 10–12% of ECs. Grade III EC showed lower expression of FGF2 than grade II EC (45). Our results showing an increased urinary abundance of FGF2 in EC patients are consistent with this. Plasma adiponectin levels are inversely associated with EC in patients aged less than 65 (46). With a mixed-age population in our study, the results show a moderate increase in adiponectin in EC patients. It could be a compensatory increase in the tumor microenvironment in response to the cancer-related metabolic stress. It also might be a result of increased shedding of the protein by the tumor tissues.

These proteomic findings are reinforced by pathway enrichment analysis. The IPA identified perturbations in complement activation, scavenger receptor-mediated uptake, and TRIM21 signaling, pathways that are increasingly recognized as central to tumor immune evasion, chronic inflammation, and altered cellular turnover in cancers. Activation of the complement system in the tumor microenvironment is reported to promote tumor growth (47). Scavenger receptors present in the tumor-associated macrophages mediate the immunosuppression within the tumor microenvironment (48). TRIM21 is usually involved in antiviral immune responses. However, recent studies indicate that TRIM21 plays a role in cancer metabolism. It is found to mediate the immune response triggered by the tumor-associated inflammation (49). The other top canonical pathway, Fc gamma receptor-dependent phagocytosis, may reflect increased antibody production, heightened innate immune surveillance, and tumor-induced inflammation in patients with EC. On the other hand, the network analysis revealed the amino acid metabolism and post-translational modification, indicating metabolic reprogramming happening in the tumor microenvironment (50). Panther classification also reflected the dysregulated proteins with several molecular functions such as catalytic activity, binding, transporter activity, and transcription regulation. The involvement of proteins in immune response, metabolic control, and cellular localization supports the hypothesis that EC exerts systemic effects beyond the endometrium, detectable through urinary profiling.

Taken together, these preliminary findings provide mechanistic insights into the urinary proteomic signature of EC. This study shows that the non-invasive biofluids, like urine, can offer valuable information on tumor-driven alterations in immunity and metabolism in patients with EC. With its non-invasive nature, we propose that urinary proteomics may serve as a platform for early detection and longitudinal monitoring. This study has several strengths. By using high-resolution LC-MS/MS-based urinary proteomics, the study paved the way for the discovery of non-invasive potential biomarker candidates for EC, a disease where early detection remains a clinical challenge. The use of urine as a biofluid provides a practical and patient-friendly sampling method, suitable for longitudinal monitoring. Moreover, the identification of novel urinary biomarkers like GLUD1, Iduronate 2-sulfatase, and histidine-rich glycoprotein with strong discriminative potential underscores the translational relevance of the findings.

While this study identifies promising urinary proteomic signatures for EC, several limitations must be acknowledged. First, the relatively small sample size may limit the generalizability of the findings and might have underpowered the multivariate analyses. The proteomic profiles were not analyzed separately for mixed disease stages and grades due to the small sample size, which may allow the few advanced cases to disproportionately influence results and introduce unaddressed biological heterogeneity. The small sample size of this pilot study precluded the analysis of medication use; however, the comparable clinical profiles suggest that any treatment-related metabolic or hypertensive influences were likely balanced across groups. The study did not include a control group with benign gynecological conditions that would have represented a more clinically relevant comparator to distinguish EC. Future studies should include a control group with benign conditions to avoid spectrum bias. We cannot rule out the possibility of the presence of any subclinical gynecological conditions in the control group. Moreover, urinary protein alterations may not always reflect systemic and transcriptomic changes. Therefore, the findings of this study should be interpreted with caution. Finally, as a discovery-phase study, the lack of an independent external validation cohort or orthogonal testing (e.g., ELISA) means these results remain preliminary. Future large-scale prospective studies are essential to validate these candidate biomarkers and confirm their translational relevance in clinical practice.

## 5 Conclusion

The study identified a preliminary urinary proteomic signature of EC. We found three key proteins, GLUD1, Iduronate 2-sulfatase, and histidine-rich glycoprotein, that were able to significantly discriminate the EC cases from controls. The dysregulated proteins, pathway enrichment analysis, and network analysis provided molecular insights into activation of Fc gamma receptor-dependent phagocytosis, complement activation, TRIM21 signaling, and amino acid metabolism, underscoring systemic immune engagement and tumor-driven metabolic shifts. These findings support the potential of urine-based profiling for EC and lay the groundwork for future validation and clinical translation.

## Data Availability

The original contributions presented in the study are publicly available. This data can be found here: http://massive.ucsd.edu/ProteoSAFe/status.jsp?task=963fae22171a4e9cb67a45f437359e5a.

## Supplementary material

Supplementary Table S1 Characteristics of study subjects.

Supplementary Table S2 Comparison of demographic and clinical parameters between endometrial cancer and controls.

Supplementary Table S3 List of significantly differentially expressed urinary proteins (DEPs) identified via LC-MS/MS, illustrating abundance changes between the endometrial cancer (EC) and control groups.

Supplementary Figure S1 Urinary proteomics pipeline for endometrial cancer biomarker identification.

Supplementary Figure S2 Distinct proteomic profiles between the control and cancer groups, unsupervised multivariate analyses **(A)** Principal Component Analysis (PCA) plot illustrates the distribution of samples based on the two principal components. **(B)** Partial Least Squares Discriminant Analysis (PLS-DA) demonstrates a clear separation between the cancer and control groups. **(C)** Orthogonal Partial Least Squares Discriminant Analysis (OPLS-DA) shows a distinct separation between the two groups, confirming a significant proteomic difference between the cancer and control conditions. The robustness of the created models was evaluated by the fitness of the model (R2Y = 0.992) and predictive ability (Q2 = 0.853) values in a larger dataset (*n* = 100).

Supplementary Figure S3 Permutation test results for model validation illustrates the results of a 100-cycle permutation test used to assess the statistical significance and stability of the OPLS-DA model. **(A)**The histograms represent the null distribution of R2Y (light blue bars) and Q2 (light red bars) generated by randomly reassigning class labels. **(B)** The original model parameters are indicated by the vertical arrows, showing a goodness of fit (R2Y) of 0.941 and a predictive ability (Q2) of 0.802. The empirical *P*-values for both metrics were *P* < 0.01 (0/100), demonstrating that the original model significantly outperforms all permuted models. These results confirm that the observed separation between the endometrial cancer (EC) and control groups is statistically robust and not a product of random chance or overfitting.
