# Supplementary material for: Label–free quantitative urinary proteomics for non-invasive biomarker discovery in endometrial cancer
Source: Front Med (Lausanne). 2026 Apr 9;13:1759839. doi: 10.3389/fmed.2026.1759839 (PMC13102663; doi:10.3389/fmed.2026.1759839)
Supplement: Supplementary Table S1 — Characteristics of study subjects. [file Table_1.docx]

**Table S1:** Characteristics of study subjects

| **Sample ID** | **Sample type** | **Age** | **BMI** | **Diagnosis** | **Stage/Grade** |
| --- | --- | --- | --- | --- | --- |
| Cancer_1 | Endometrial Cancer | 70 | 33.69 | Endometrioid endometrial adenocarcinoma | 1A/G2 |
| Cancer_2 | Endometrial Cancer | 60 | 48.50 | Endometrioid endometrial adenocarcinoma | 1A/G2 |
| Cancer_ 3 | Endometrial Cancer | 60 | 48.00 | Endometrioid endometrial adenocarcinoma | 1A/G1 |
| Cancer_ 4 | Endometrial Cancer | 45 | 29.90 | Endometrioid endometrial adenocarcinoma | 1B/G3 |
| Cancer_ 5 | Endometrial Cancer | 50 | 32.00 | Endometrioid endometrial adenocarcinoma | 1B/G1 |
| Cancer_ 6 | Endometrial Cancer | 68 | 27.89 | Endometrioid endometrial adenocarcinoma | 3C/G3 |
| Cancer_ 7 | Endometrial Cancer | 50 | 31.00 | Endometrioid endometrial adenocarcinoma | 3C/G2 |
| Cancer_ 8 | Endometrial Cancer | 72 | 25.36 | Endometrioid endometrial adenocarcinoma | 3A/G2 |
| Cancer_ 9 | Endometrial Cancer | 59 | 45.61 | Endometrioid endometrial adenocarcinoma | 1A/G3 |
| Cancer_10 | Endometrial Cancer | 54 | 30.00 | Endometrioid endometrial adenocarcinoma | 1A/G2 |
| Cancer_11 | Endometrial Cancer | 58 | 44.23 | Endometrioid endometrial adenocarcinoma | 1A/G1 |
| Cancer_12 | Endometrial Cancer | 52 | 35.02 | Endometrioid endometrial adenocarcinoma | 1A/G1 |
| Cancer_13 | Endometrial Cancer | 55 | 44.06 | Endometrioid endometrial adenocarcinoma | 1A/G1 |
| Cancer_14 | Endometrial Cancer | 62 | 29.36 | Endometrioid endometrial adenocarcinoma | 1B/G1 |
| Cancer_15 | Endometrial Cancer | 67 | 33.56 | Endometrioid endometrial adenocarcinoma | 1A/G2 |
| Cancer_16 | Endometrial Cancer | 63 | 37.25 | Endometrioid endometrial adenocarcinoma | 1A/G3 |
| Cancer_17 | Endometrial Cancer | 59 | 38.00 | Endometrioid endometrial adenocarcinoma | 1A/G2 |
| Cancer_18 | Endometrial Cancer | 58 | 34.01 | Endometrioid endometrial adenocarcinoma | 1A/G3 |
| Cancer_ 19 | Endometrial Cancer | 62 | 46.28 | Endometrioid endometrial adenocarcinoma | 1A/G2 |
| Cancer_20 | Endometrial Cancer | 66 | 35.98 | Endometrioid endometrial adenocarcinoma | 1A/G2 |
| C 1 | Control | 48 | 28.21 | Non-Endometrial Cancer |  |
| C2 | Control | 43 | 39.89 | Non-Endometrial Cancer |  |
| C3 | Control | 63 | 41.62 | Non-Endometrial Cancer |  |
| C 4 | Control | 44 | 46.93 | Non-Endometrial Cancer |  |
| C 5 | Control | 65 | 30.02 | Non-Endometrial Cancer |  |
| C6 | Control | 52 | 24.90 | Non-Endometrial Cancer |  |
| C7 | Control | 52 | 23.70 | Non-Endometrial Cancer |  |
| C8 | Control | 52 | 23.30 | Non-Endometrial Cancer |  |
| C9 | Control | 43 | 39.90 | Non-Endometrial Cancer |  |
| C10 | Control | 43 | 39.80 | Non-Endometrial Cancer |  |
| C11 | Control | 40 | 39.02 | Non-Endometrial Cancer |  |
| C12 | Control | 43 | 29.20 | Non-Endometrial Cancer |  |
| C13 | Control | 47 | 33.02 | Non-Endometrial Cancer |  |
| C14 | Control | 52 | 35.23 | Non-Endometrial Cancer |  |
| C15 | Control | 58 | 28.00 | Non-Endometrial Cancer |  |
| C16 | Control | 62 | 37.89 | Non-Endometrial Cancer |  |
| C17 | Control | 70 | 35.00 | Non-Endometrial Cancer |  |
| C18 | Control | 71 | 39.00 | Non-Endometrial Cancer |  |
| C19 | Control | 66 | 30.00 | Non-Endometrial Cancer |  |
| C20 | Control | 69 | 35.00 | Non-Endometrial Cancer |  |
