# Supplementary material for: Label–free quantitative urinary proteomics for non-invasive biomarker discovery in endometrial cancer
Source: Front Med (Lausanne). 2026 Apr 9;13:1759839. doi: 10.3389/fmed.2026.1759839 (PMC13102663; doi:10.3389/fmed.2026.1759839)
Supplement: Supplementary Table S2 — Comparison of demographic and clinical parameters between endometrial cancer and controls. [file Table_2.docx]

**Table S2.** Comparison of demographic and clinical parameters between EC patients and controls

|  | **Control (n=20)** | **EC**  **(n=20)** | **P value** |
| --- | --- | --- | --- |
| Age (years) | 54.15±10.45 | 59.50 ±7.13 | 0.066 |
| BMI (kg/m^2^) | 33.98±6.59 | 36.49±7.20 | 0.259 |
| SBP (mm Hg) | 126.04±18.88 | 131.50±14.43 | 0.307 |
| DBP(mm Hg) | 68.52±12.61 | 66.35±9.23 | 0.534 |
| Serum creatinine (µmol/L) | 63.35±9.84 | 64.05±15.01 | 0.818 |
| BUN (mmol/L) | 3.80±1.49 | 4.45±1.74 | 0.210 |
| eGFR (ml/min/1.73m²) | 98.70±12.45 | 89.50±16.95 | 0.072 |
| FBG (mmol/L) | 7.23±3.25 | 7.88±3.42 | 0.535 |
| HbA1c (%) | 7.88±3.42 | 6.05±1.00 | 0.093 |

EC, endometrial cancer; BMI, body mass index; SBP, systolic blood pressure; DBP, diastolic blood pressure; BUN, blood urea nitrogen; eGFR, estimated glomerular filtration rate; FBG, fasting blood glucose. Data are presented as mean ± standard deviation and compared by Student T test. Values of *P < 0.05 were considered significant.
