# Supplementary figures and images for: Label–free quantitative urinary proteomics for non-invasive biomarker discovery in endometrial cancer
Source: Front Med (Lausanne). 2026 Apr 9;13:1759839. doi: 10.3389/fmed.2026.1759839 (PMC13102663; doi:10.3389/fmed.2026.1759839)

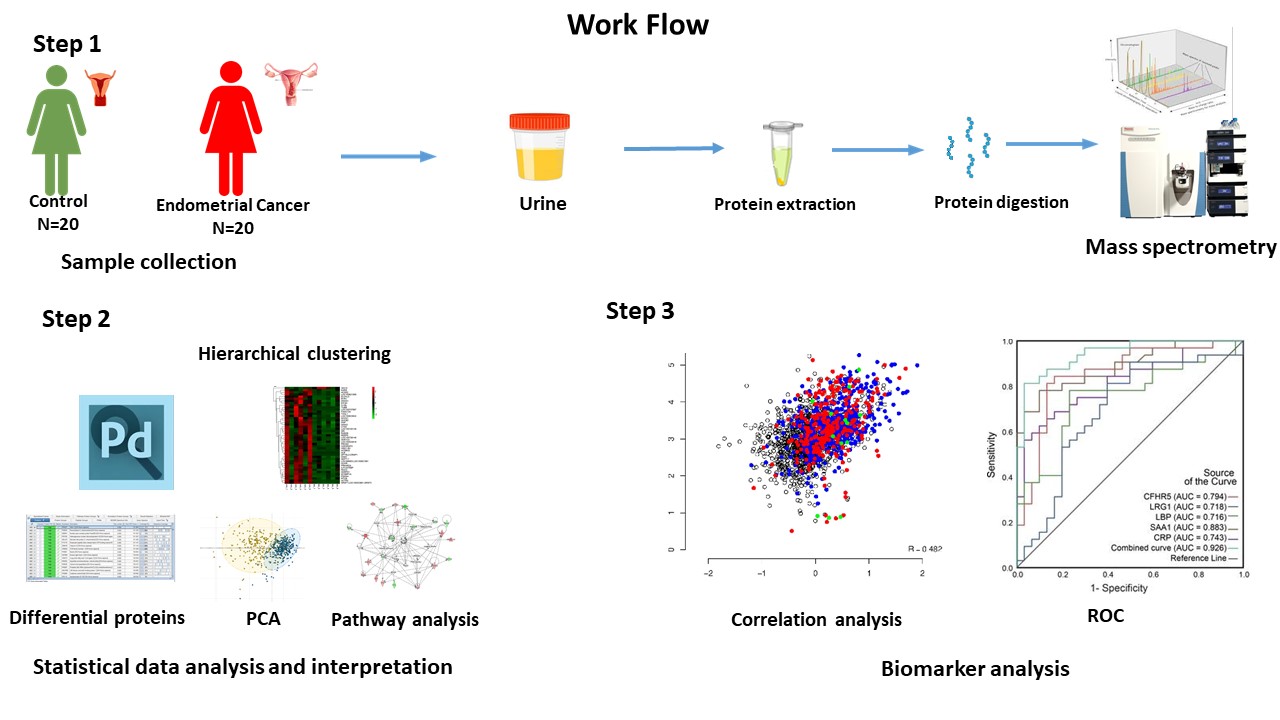

Supplement: Supplementary Figure S1 — Urinary proteomics pipeline for endometrial cancer biomarker identification. [file Image_1.jpeg]

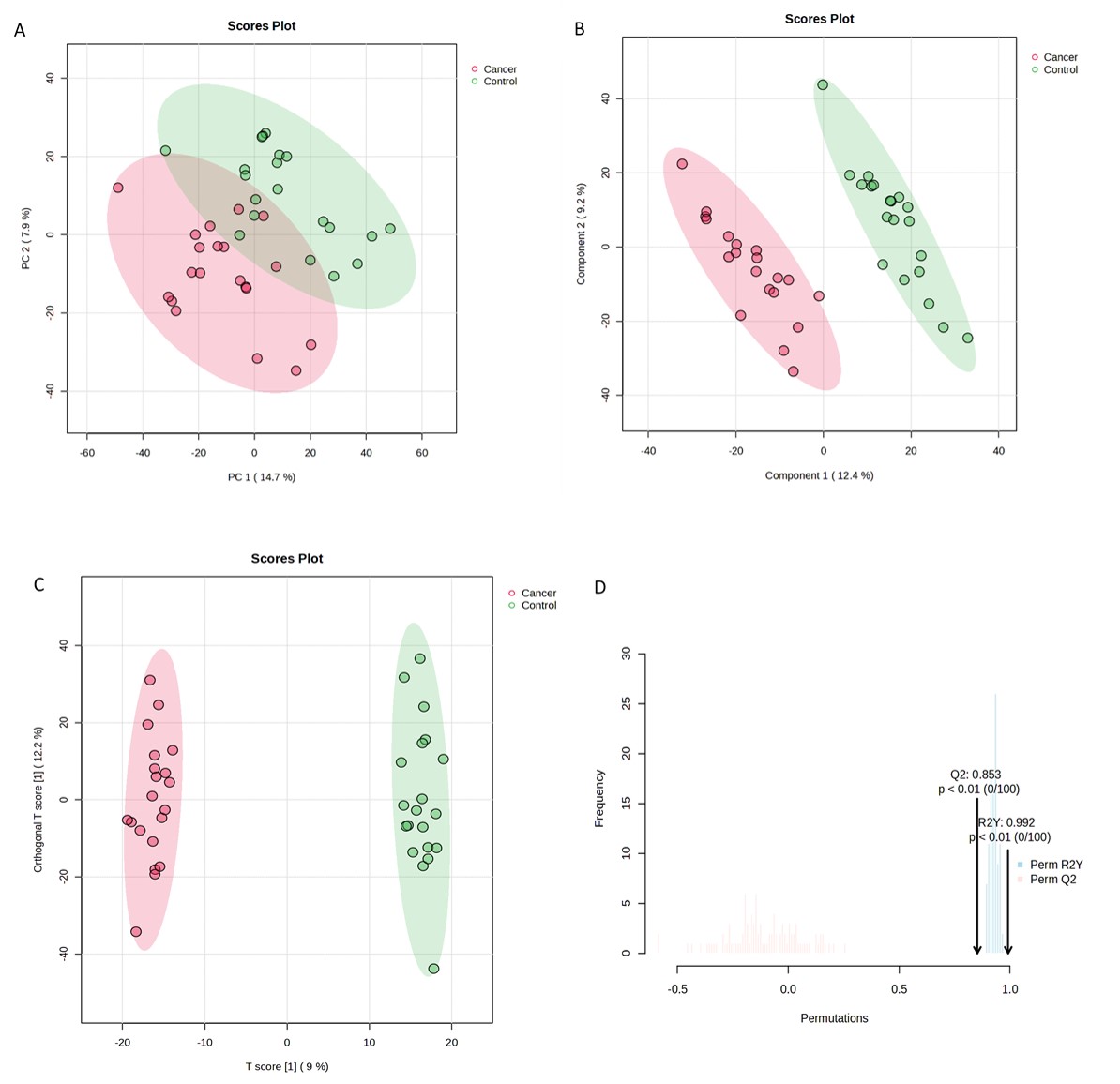

Supplement: Supplementary Figure S2 — Distinct proteomic profiles between the control and cancer groups, unsupervised multivariate analyses (A) Principal Component Analysis (PCA) plot illustrates the distribution of samples based on the two principal components. (B) Partial Least Squares Discriminant Analysis (PLS-DA) demonstrates a clear separation between the cancer and control groups. (C) Orthogonal Partial Least Squares Discriminant Analysis (OPLS-DA) shows a distinct separation between the two groups, confirming a significant proteomic difference between the cancer and control conditions. The robustness of the created models was evaluated by the fitness of the model (R2Y = 0.992) and predictive ability (Q2 = 0.853) values in a larger dataset (n = 100). [file Image_2.jpeg]

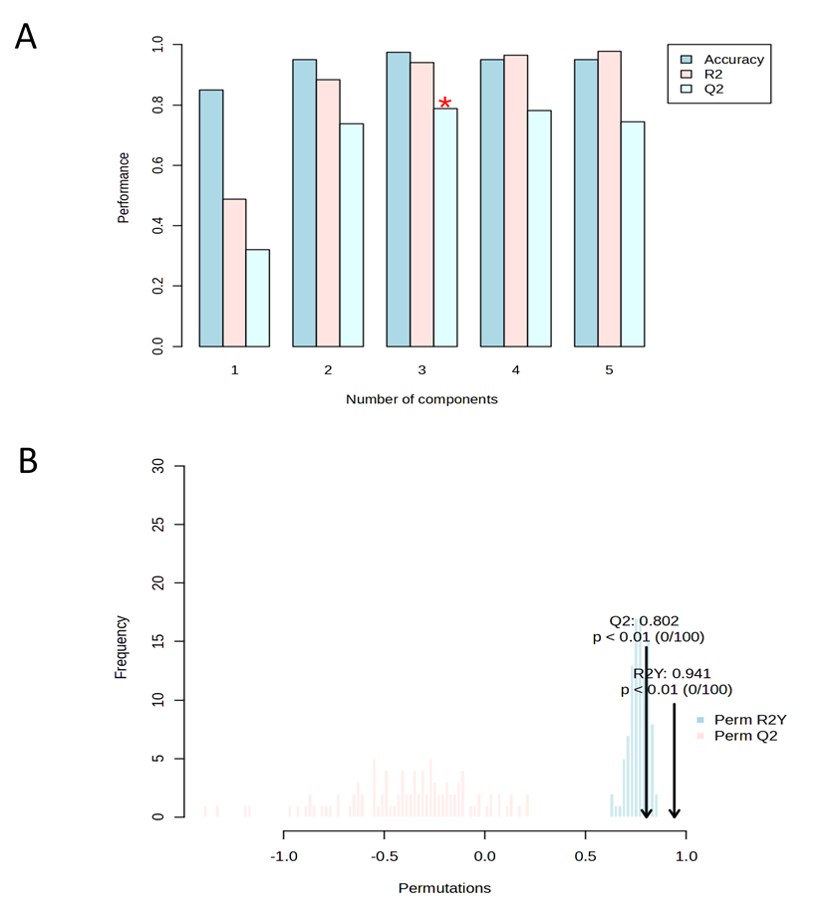

Supplement: Supplementary Figure S3 — Permutation test results for model validation illustrates the results of a 100-cycle permutation test used to assess the statistical significance and stability of the OPLS-DA model. (A)The histograms represent the null distribution of R2Y (light blue bars) and Q2 (light red bars) generated by randomly reassigning class labels. (B) The original model parameters are indicated by the vertical arrows, showing a goodness of fit (R2Y) of 0.941 and a predictive ability (Q2) of 0.802. The empirical P-values for both metrics were P < 0.01 (0/100), demonstrating that the original model significantly outperforms all permuted models. These results confirm that the observed separation between the endometrial cancer (EC) and control groups is statistically robust and not a product of random chance or overfitting. [file Image_3.jpeg]
